# Diurnal Insulin Clearance and Circadian Metabolic Gene Signatures in MASLD: Integrative Multi-Dataset Physiological and Transcriptomic Analysis

**DOI:** 10.3390/metabo16090629

**Published:** 2026-08-29

**Authors:** Lin Guo, Yimin Yin, Yanyan Sun, Hongwen Zhou, Yingyun Gong

**Affiliations:** Department of Endocrinology, The First Affiliated Hospital with Nanjing Medical University, Nanjing 210029, China; gl2024220109@stu.njmu.edu.cn (L.G.); yym@stu.njmu.edu.cn (Y.Y.); syy260713@stu.njmu.edu.cn (Y.S.)

**Keywords:** insulin clearance, diurnal variation, MASLD, circadian rhythm, insulin resistance, hepatic metabolism, spatial transcriptomics

## Abstract

**Highlights:**

**What are the main findings?**

- Insulin clearance showed a diurnal pattern across a single-subject pilot assessment and independent clamp datasets, with relatively higher evening/nighttime clearance.
- Multi-dataset integration identified insulin clearance-related and circadian metabolic gene signatures altered in MASLD liver, spatial transcriptomic, and high fat diet (HFD)-induced mouse datasets.

**What are the implications of the main findings?**

- Insulin clearance may be a time-dependent metabolic phenotype linked to nocturnal metabolic vulnerability in MASLD.
- These findings support future controlled human and mechanistic studies on circadian regulation of insulin clearance in MASLD.

**Abstract:**

Background/Objectives: Insulin clearance is a key determinant of circulating insulin availability, but its diurnal variation and relationship with circadian metabolic programs in metabolic dysfunction associated steatotic liver disease (MASLD) remain unclear. This study aimed to explore diurnal insulin clearance in humans and examine associated metabolic gene signatures in MASLD. Methods: A single-subject pilot assessment was performed to explore daytime-nighttime differences in insulin clearance rate (ICR) surrogate index, followed by evaluation using public hyperinsulinemic-euglycemic clamp datasets from healthy individuals and patients with MASLD. Public circadian transcriptomic datasets, spatial transcriptomic data, and a time course high-fat diet (HFD)-induced mouse dataset were integrated. A predefined panel of insulin clearance-related and circadian genes, including carcinoembryonic antigen-related cell adhesion molecule 1 (*CEACAM1*), insulin receptor (*INSR*), insulin-degrading enzyme (*IDE*), clock circadian regulator (*CLOCK*), basic helix-loop-helix ARNT like 1 (*BMAL1*), nuclear receptor subfamily 1 group D member 1/2 (*NR1D1/2*), period circadian regulator 1/2 (*PER1/2*), and cryptochrome 1/2 (*CRY1/2*), was analyzed. Results: The pilot assessment showed higher nighttime than daytime ICR, and independent clamp datasets showed a similar pattern in healthy individuals. In MASLD, nighttime ICR remained relatively higher, whereas overall insulin clearance was reduced compared with controls. Human blood-based circadian transcriptomic datasets identified rhythmic expression patterns of selected genes involved in insulin clearance and circadian regulation, including *CEACAM1*, *CLOCK*, *NR1D1*, *CRY1*, *PER1*, and *PER2*. MASLD liver datasets showed reduced expression of insulin clearance-related and circadian genes, while spatial transcriptomics suggested altered lobular distribution of these signatures. In HFD mouse model, rhythmic expression of selected genes was attenuated. Conclusions: These integrative findings suggest that insulin clearance may exhibit diurnal variation and may be linked to circadian metabolic gene signatures across systemic and hepatic datasets in MASLD. Larger controlled human studies are needed to validate the temporal regulation of insulin clearance and its metabolic relevance.

## 1. Introduction

Insulin homeostasis is maintained through the coordinated regulation of insulin secretion, insulin action, and insulin clearance. Among these processes, hepatic insulin clearance is a major determinant of systemic insulin availability, as the liver removes a substantial proportion of endogenously secreted insulin during its first pass through the portal circulation [1]. Impaired insulin clearance may contribute to chronic hyperinsulinemia, insulin resistance, and metabolic dysfunction, thereby amplifying pathogenic feedback loops across insulin-sensitive tissues. Hepatic receptor-mediated insulin uptake and degradation involve several molecular components, including insulin receptor signaling, carcinoembryonic antigen-related cell adhesion molecule 1 (CEACAM1) [2,3], and insulin-degrading enzyme (IDE) [1], supporting the central role of the liver in systemic insulin metabolism.

Circadian rhythms are fundamental regulators of metabolic homeostasis. A hierarchical clock system [4], composed of the central pacemaker in the suprachiasmatic nucleus and peripheral clocks in metabolic organs such as the liver [5], coordinates daily oscillations in glucose, lipid, and energy metabolism [6]. Disruption of circadian organization has been associated with obesity, insulin resistance, type 2 diabetes, and liver metabolic dysfunction [7]. Recent clinical studies [8] have further highlighted pronounced time-of-day variation in metabolic dysfunction–associated steatotic liver disease (MASLD), including alterations in hepatic and peripheral insulin sensitivity, de novo lipogenesis, substrate utilization, and circulating insulin availability during the nighttime or resting phase.

These observations raise the possibility that insulin clearance may represent a dynamic, time-dependent component of metabolic regulation rather than a static hepatic function. In MASLD [8], reduced nocturnal insulin availability has been linked to both altered insulin secretion and elevated insulin clearance, suggesting that temporal dysregulation of insulin metabolism may contribute to disease-related metabolic imbalance. However, the molecular correlates of diurnal insulin clearance remain insufficiently characterized. It remains unclear whether insulin clearance-related genes, such as *CEACAM1*, insulin receptor (*INSR*), and *IDE*, are temporally coordinated with core circadian regulators, including clock circadian regulator (*CLOCK*), basic helix-loop-helix ARNT like 1 (*BMAL1*), nuclear receptor subfamily 1 group D member 1/2 (*NR1D1/2*), period circadian regulator 1/2 (*PER1/2*), and cryptochrome 1/2 (*CRY1/2*) and whether these circadian metabolic signatures are altered in the MASLD liver.

Direct high-resolution assessment of hepatic circadian programs in humans remains challenging because repeated liver sampling across the day–night cycle is rarely feasible [6]. Integrative analysis of physiological phenotyping, public systemic circadian transcriptomic datasets, disease-associated liver expression profiles, spatial transcriptomics, and experimental model datasets may therefore provide a useful hypothesis-generating framework [9]. Such an approach can help connect diurnal metabolic phenotypes with candidate hepatic gene signatures and the spatial organization of metabolic programs within the liver microenvironment.

In the present study, we explored diurnal insulin clearance using a single-subject pilot assessment and independent public hyperinsulinemic-euglycemic clamp (HEC) datasets from healthy individuals and patients with MASLD. We further integrated public circadian transcriptomic datasets, MASLD liver expression datasets, spatial transcriptomic data, and a time course HFD mouse model to examine insulin clearance-related and circadian gene signatures. We hypothesized that diurnal variation in insulin clearance may be associated with hepatic circadian metabolic gene programs and that these signatures may be altered in MASLD.

## 2. Materials and Methods

### 2.1. Single-Subject Pilot Assessment

A single-subject pilot assessment was conducted in a 29-year-old female with a body mass index of 20.1 kg/m^2^ and stable body weight. The tests were performed on the same day, with an overnight fast prior to the morning assessment, while an 8 h fast was maintained prior to the evening assessment. The participant had no history of metabolic disease or chronic illness. Exclusion criteria included diabetes, dyslipidemia, chronic liver disease, sleep disorders, shift work within the previous 6 months, regular alcohol consumption (>14 drinks/week), and the use of medications known to affect insulin metabolism or circadian rhythms. The participant reported a regular menstrual cycle and denied the use of hormonal contraceptives, pregnancy, or breastfeeding. Written informed consent was obtained from the participant. The study protocol was approved by the Institutional Review Board (2018-SR-069) and was conducted in accordance with the Declaration of Helsinki.

### 2.2. Sleep–Wake Schedule and Dietary Standardization

The participant was instructed to follow a standardized sleep–wake schedule for 7 days before metabolic testing, with scheduled sleep from 23:00 to 07:00 and wakefulness from 07:00 to 23:00. Regular sleep patterns were self-reported and further supported by wearable device-based sleep monitoring data collected during the preceding 2 weeks. Dietary intake was standardized by providing dietary recommendations based on an approximate macronutrient composition of 55% carbohydrate, 25% fat, and 20% protein. During the pre-assessment period, meals were consumed at a fixed location to improve consistency in dietary exposure.

### 2.3. Steamed Bread Meal Test and Estimation of Insulin Clearance

A pilot daytime–evening comparison of a meal test-derived insulin clearance surrogate index was performed at two predefined Zeitgeber time (ZT) points: ZT0, corresponding to 07:00 and scheduled wake onset, and ZT12, corresponding to 19:00 and the evening time point. At ZT0, the test was performed after an overnight fast. At ZT12, the participant fasted for 8 h before testing, with water intake allowed.

At each time point, a 2 h steamed bread meal test was performed using a 100 g steamed bread meal as a standardized carbohydrate challenge. Blood samples were collected at 0, 30, 60, and 120 min for measurement of plasma glucose, insulin, and C-peptide concentrations. All biochemical measurements were performed by the clinical laboratory of the First Affiliated Hospital with Nanjing Medical University (Nanjing, China) according to routine standardized procedures. Total area under the curve values from 0 to 120 min were calculated using the trapezoidal method.

A meal test-derived insulin clearance surrogate index was calculated using the molar ratio of total C-peptide area under the curve (AUC) to total insulin AUC [10]:

ICR-surrogate index = CpAUC120/InsAUC120.

Insulin and C-peptide concentrations were converted to molar units before calculation. This index was interpreted as a surrogate marker reflecting relative insulin clearance capacity during the meal test and should not be considered equivalent to direct hepatic insulin extraction or clamp-derived insulin metabolic clearance rate.

### 2.4. Public HEC Datasets

To complement the meal test-derived surrogate estimate with a secretion-independent assessment, publicly available HEC datasets from healthy individuals and patients with MASLD were analyzed. Controls and MASLD patients were 6 per group and matched within subjects for daytime and nighttime measurements. Hyperinsulinemic euglycemic clamp started at 07:00 AM during the day and 19:00 PM during the night. [U-13C]-glucose (2 mg/kg) was administered as a bolus over 1 min followed by a continuous infusion of 0.9% saline (20 μg/kg/min). Blood glucose was monitored at 15 min intervals during the 120 min. Prehepatic insulin secretion rate was calculated from deconvolution of plasma C-peptide concentrations.

For clamp-derived insulin clearance, the metabolic clearance rate of insulin was calculated as the steady-state insulin infusion rate divided by the steady-state plasma insulin concentration [11]:

CLwb = steady-state insulin infusion rate/steady-state plasma insulin concentration.

We used pooled estimates reported in publications for statistical analysis.

### 2.5. Public Transcriptomic and Spatial Datasets

Publicly available transcriptomic datasets were obtained from the Gene Expression Omnibus database. The accession numbers, species, tissue or cell type, study design, sampling time points, platform, sample size, and primary analytical use of each dataset are provided in Supplementary Table S1. All analyses were performed using publicly available de-identified datasets, and no additional ethical approval was required for secondary analysis of these datasets.

The human liver dataset GSE126848 was used to assess disease-associated gene expression differences between individuals with MASLD and healthy controls. Because repeated 24 h liver biopsies in healthy humans are ethically and logistically infeasible, two human peripheral blood time-series transcriptomic datasets were analyzed as systemic circadian proxy datasets: GSE220120, consisting of white blood cell transcriptomic profiles collected every 2 h across a 24 h period, and GSE122541, consisting of peripheral blood mononuclear cell transcriptomic profiles collected every 3 h across a 24 h period. These datasets were used to examine time-dependent expression patterns of predefined insulin clearance–related and circadian genes. Because these datasets were derived from peripheral blood rather than liver tissue, the results were interpreted as systemic circadian signatures and not as direct hepatic circadian expression profiles.

The dataset GSE278920 was used to evaluate time-dependent hepatic gene expression in CD and 60% HFD mouse models sampled across multiple circadian time points. This dataset was used to assess whether insulin clearance-related and circadian gene signatures were altered under metabolic stress in liver tissue.

Spatial transcriptomic data from human MASLD liver tissue were analyzed to examine the spatial distribution of insulin clearance–related and circadian gene signatures within the liver microenvironment. Dataset accession numbers, preprocessing methods, spatial resolution, quality-control criteria, normalization procedures, and regional annotation strategies are described in Supplementary Table S1.

### 2.6. Gene Selection

A predefined panel of genes involved in insulin clearance and circadian regulation was selected based on prior biological relevance. The insulin clearance–related genes included *CEACAM1*, *INSR*, and *IDE*. The circadian regulatory genes included *CLOCK*, *BMAL1*, *NR1D1*, *NR1D2*, *PER1*, *PER2*, *CRY1* and *CRY2*. This focused candidate-gene strategy was used to reduce dimensionality and to align the transcriptomic analyses with the physiological phenotype of interest.

### 2.7. Data Processing

Gene expression data were processed using R version 4.2.2. For microarray datasets, background correction, quantile normalization, and log2 transformation were performed using the limma package when raw data were available. For RNA-sequencing datasets, normalized expression matrices provided by the original studies were used when raw count data or full metadata were unavailable. Probe identifiers were mapped to official gene symbols. When multiple probes mapped to the same gene, the probe with the highest average expression across samples was retained. Quality-control procedures, normalization approaches, and batch-correction strategies were applied according to dataset type.

### 2.8. Statistical Analysis

Statistical analyses were performed using GraphPad Prism version 8.0.2 and R version 4.2.2. For the single-subject pilot assessment, results were treated as exploratory and descriptive, without inferential statistical testing. For group comparisons in public datasets, data normality was assessed using the Shapiro–Wilk test when applicable. Paired or unpaired two-tailed tests were used according to the structure of each dataset.

For transcriptomic datasets, differential gene expression between groups was assessed using limma or the statistical framework appropriate for the original data type. Circadian rhythmicity and rhythmic parameters, including MESOR, amplitude, and phase radians, were evaluated using the circacompare R package under a fixed 24 h period model [12]. To reduce false-positive findings in multi-gene analyses, the Benjamini–Hochberg false discovery rate procedure was applied to transcriptomic differential expression analyses and rhythmicity tests. An FDR-adjusted q-value < 0.05 was considered statistically significant.

### 2.9. Data, Code, and Protocol Availability

All public datasets analyzed in this study are available from the Gene Expression Omnibus or the original public repositories under the accession numbers listed in Supplementary Table S1. Processed data matrices, analysis scripts, and the analytical protocol used to generate the main results and figures will be made available through a public repository before publication or upon reasonable request during peer review. Any restrictions on access to individual-level clinical or pilot participant data will be disclosed at submission in accordance with institutional and ethical requirements.

## 3. Results

### 3.1. Diurnal Variation in Insulin Clearance in Humans

**Figure 1:**
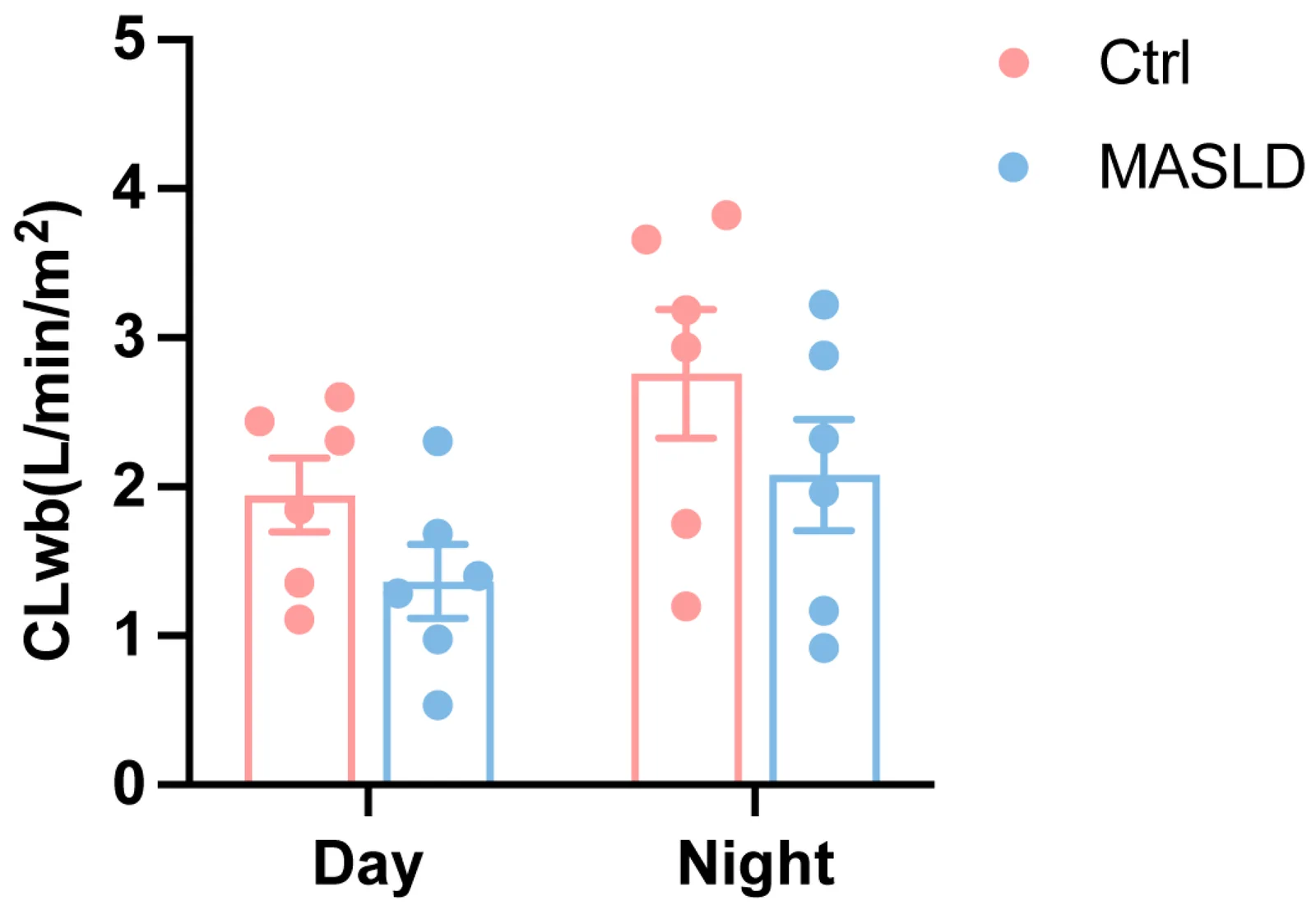
Diurnal variation in insulin clearance rate using hyperinsulinemic-euglycemic clamp datasets in control subjects and MASLD patients. Data are presented as mean ± SEM. Source data is adapted from Cell Metabolism, 2026; 38, 474–492.e6 [8]. Ctrl: control subject, MASLD: MASLD patients, CLwb: Whole-body insulin clearance during the clamp.

The meal test-derived insulin clearance surrogate index was numerically higher at the evening measurement (11.08) compared with the morning measurement (6.88) in the single-subject pilot assessment. To examine whether a similar time-of-day pattern was observed in independent clamp data, we analyzed independent HEC datasets. Clamp-derived insulin metabolic clearance rate showed a similar daytime–nighttime pattern in independent datasets, whereas overall insulin clearance capacity differed between individuals with MASLD and controls (Figure 1), indicating impaired insulin clearance capacity in metabolic disease.

### 3.2. Circadian Gene Expression Patterns Associated with Insulin Clearance

To explore potential regulatory mechanisms, we analyzed public human transcriptomic datasets (GSE220120 and GSE122541) and identified significant circadian rhythmicity in genes involved in insulin clearance and clock regulation.

We selected a panel of eleven core genes involved in hepatic insulin clearance as well as circadian rhythms: *CEACAM1*, *INSR*, *IDE*, *CLOCK*, *BMAL1 (ARNTL)*, *NR1D1 (REV-ERBα)*, *NR1D2 (REV-ERBβ)*, *PER1*, *PER2*, *CRY1* and *CRY2* for analysis and found that six of the eleven selected genes showed significant circadian rhythmicity in the dataset. Peripheral blood *CEACAM1* level, a core gene involved in insulin clearance, reached a trough at ZT6 (0.31 ± 0.17) and a peak at ZT18 (1.22 ± 0.43); levels of peripheral blood *CLOCK*, a circadian core gene, reached a trough at ZT9 (168.6 ± 10.1) and a peak at ZT21 (201.3 ± 11.2); whereas *INSR*, *IDE*, *NR1D2*, *CRY2* and *BMAL1* did not show consistent rhythmic expression.

**Figure 2:**
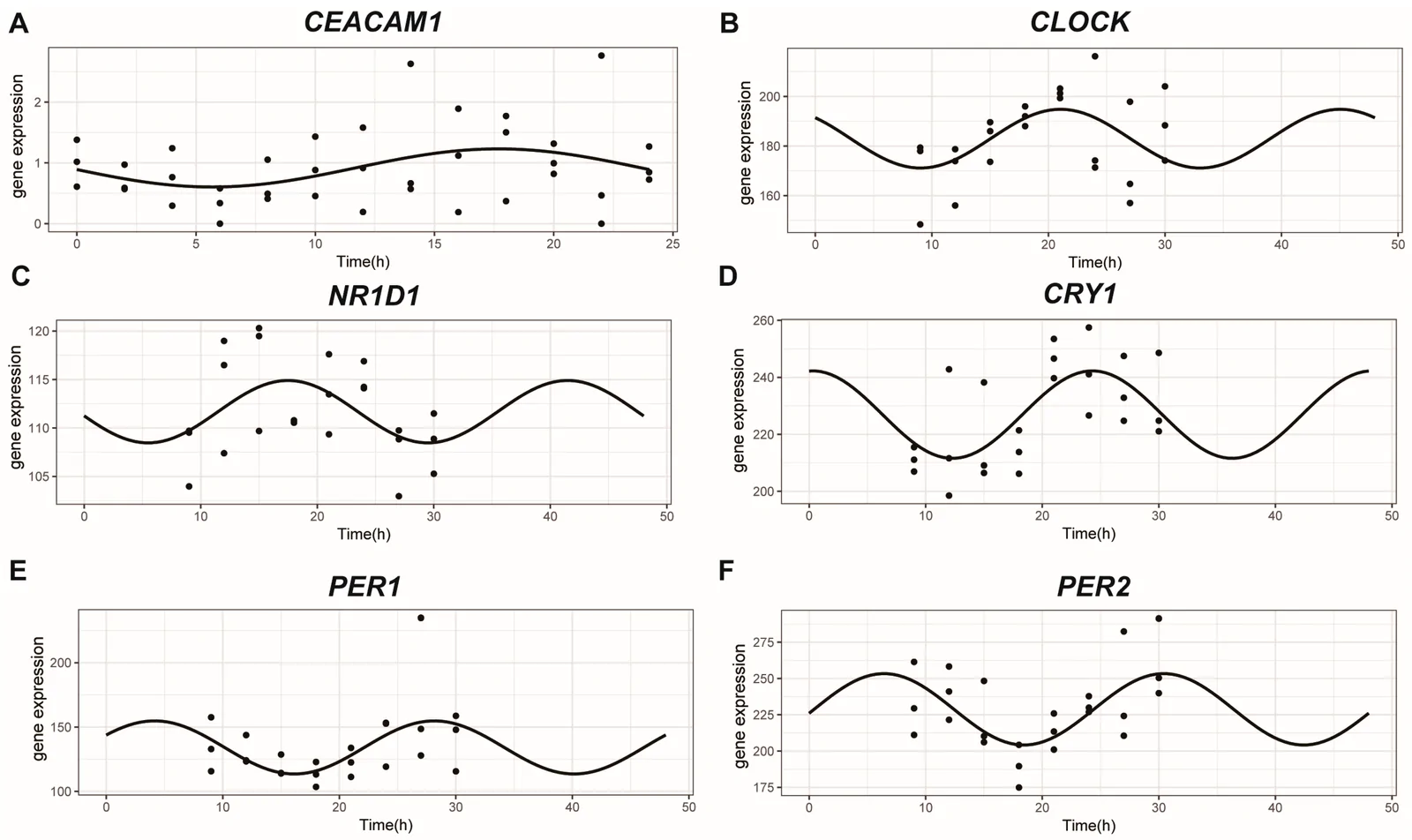
Circadian expression patterns of insulin clearance-related and clock genes. Circadian rhythmicity of genes involved in insulin clearance and clock regulation in public transcriptomic datasets (*CEACAM1* from GSE220120, and the others from GSE122541). Genes exhibiting significant rhythmicity include *CEACAM1* (**A**), *CLOCK* (**B**), *NR1D1* (**C**), *CRY1* (**D**), *PER1* (**E**), and *PER2* (**F**), with distinct phase patterns. Rhythmicity was assessed using cosinor-based analysis. Only genes with statistically significant oscillations are shown [12].

The rhythmic genes could be divided into two clusters based on phase alignment:

Active phase-peaking genes: *CEACAM1* (Figure 2A), *CLOCK* (Figure 2B). These genes peaked at ZT17-ZT22, consistent with the peak of ICR. *CEACAM1* exhibited the highest amplitude (3.94-fold change between peak and trough), followed by *NR1D1* (1.05-fold).

Resting phase-peaking genes: *CRY1* (Figure 2D), *PER1* (Figure 2E), and *PER2* (Figure 2F). These genes peaked at ZT0–ZT6, with a distinct temporal expression pattern compared with ICR and the active phase cluster.

### 3.3. Spatial Variations in Insulin Clearance-Related Pathways in MASLD

**Figure 3:**
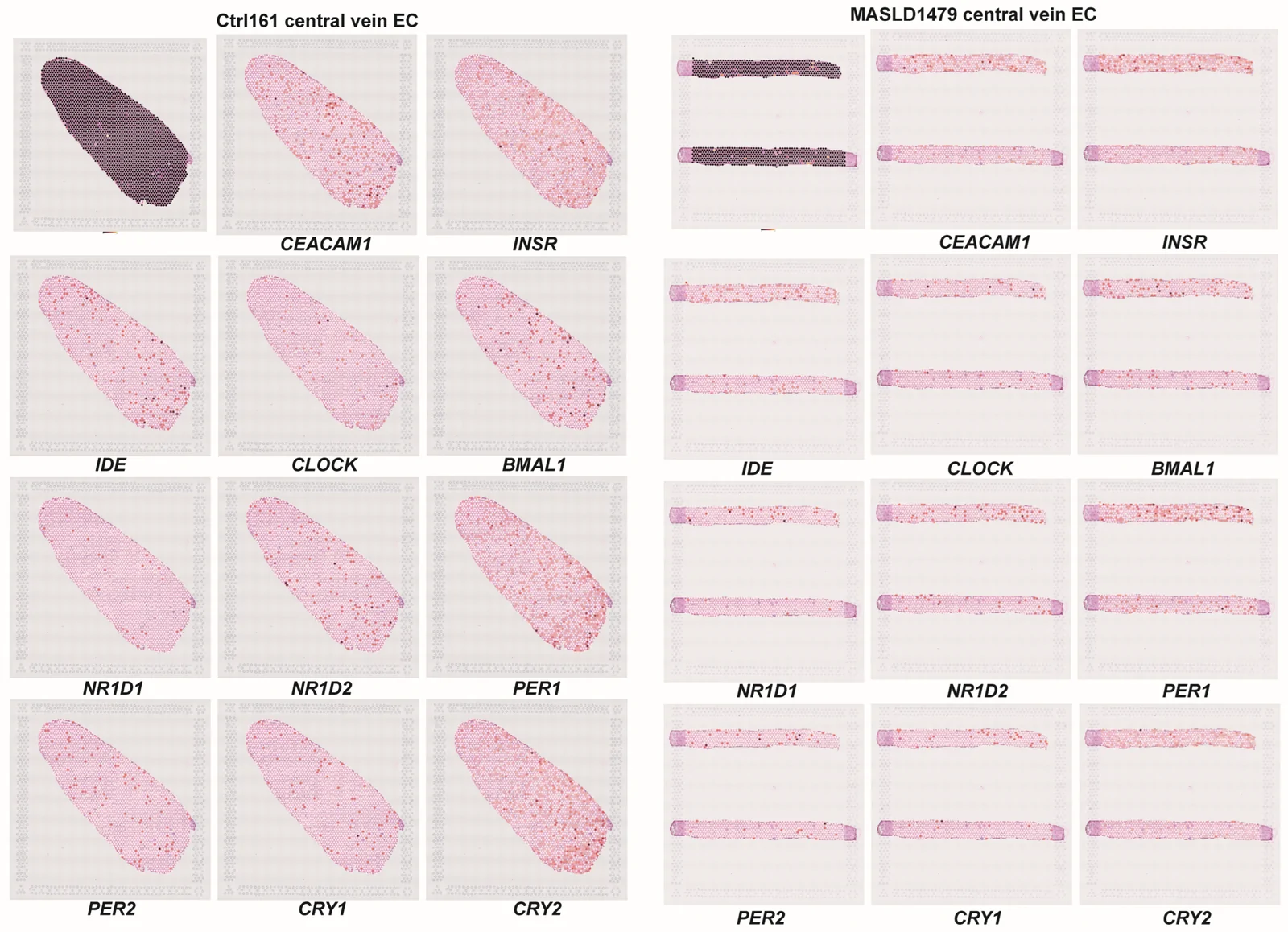
Spatial variations in insulin clearance-related pathways in MASLD. Spatial transcriptomic analysis showing the distribution of insulin clearance-related and circadian genes in liver tissue from healthy controls (**left panel**) and MASLD patients (**right panel**). The first picture of the representative map of healthy control (**left panel**) and MASLD patient (**right panel**) reflects the overall gene distribution, and the remaining pictures reflect the distribution of the corresponding genes. Reduced expression of these genes is observed in the central lobular region in MASLD compared with controls, indicating altered spatial organization of metabolic pathways. Representative spatial maps are shown.

Spatial transcriptomics is able to delineate the spatial distribution of RNA within individual tissue sections and provides valuable insights into the development of MASLD [13]. To further investigate the spatial organization of insulin clearance-related pathways, we analyzed spatial transcriptomic datasets from healthy individuals and MASLD patients [9]. We observed changes in expression of genes involved in insulin clearance and circadian regulation in the central lobular region of the liver in MASLD compared with healthy controls (Figure 3).

These findings indicate that metabolic stress alters not only gene expression levels but also the spatial organization of circadian metabolic pathways within the hepatic lobule.

### 3.4. Dysregulation of the Insulin Clearance–Circadian Axis in MASLD

**Figure 4:**
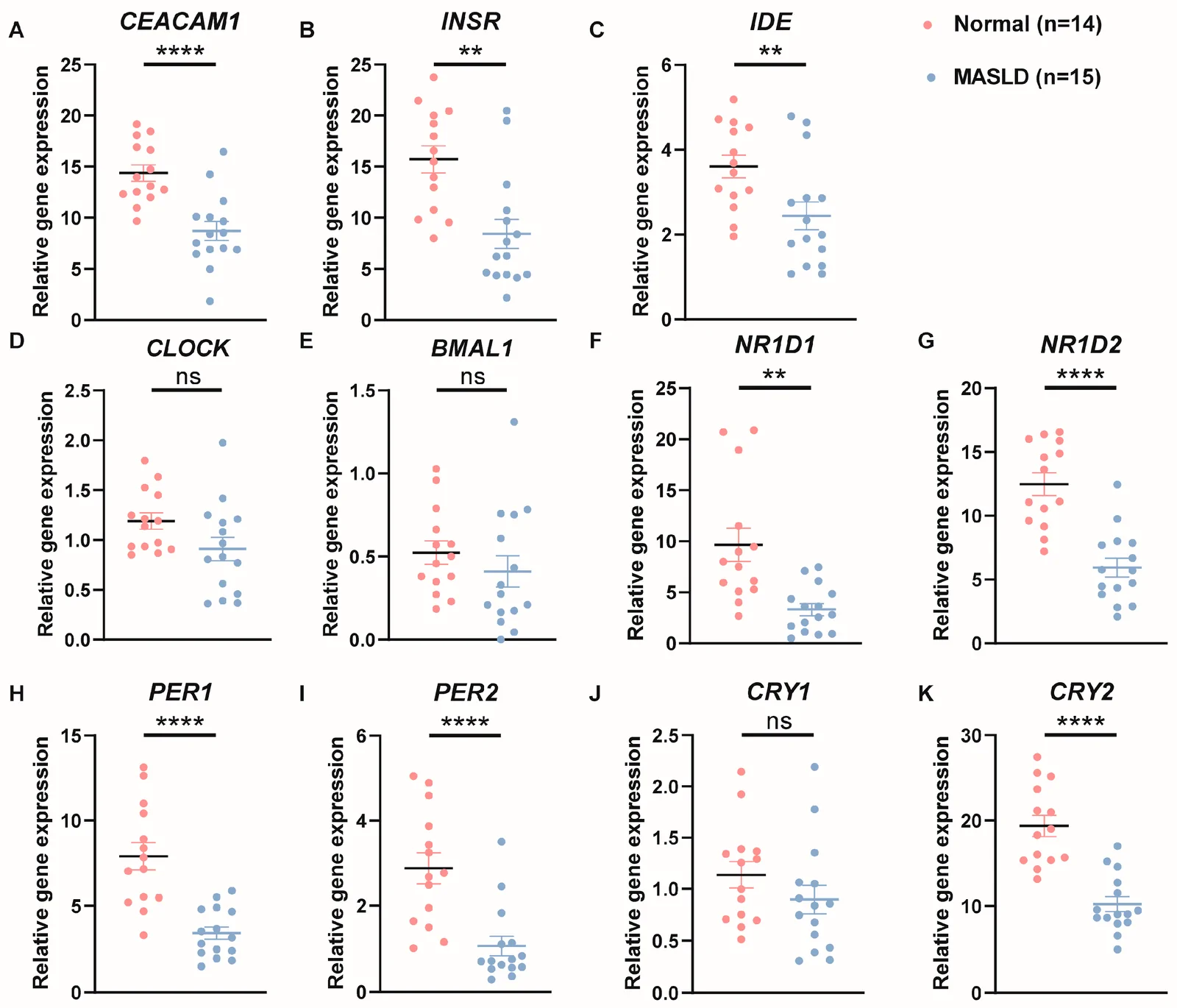
Dysregulation of insulin clearance and circadian genes in MASLD (GSE126848). Expression levels of insulin clearance–related genes *CEACAM1* (**A**), *INSR* (**B**), *IDE* (**C**) and circadian clock genes *CLOCK* (**D**), *BMAL1* (**E**), *NR1D1* (**F**), *NR1D2* (**G**), *PER1* (**H**), *PER2* (**I**), *CRY1* (**J**), *CRY2* (**K**) in liver transcriptomic datasets from MASLD patients and controls. Data are presented as mean ± SEM. Statistical comparisons were performed between groups. ns FDR-adjusted q-value > 0.05, ** q < 0.01, **** q < 0.0001.

We next assessed whether the insulin clearance–circadian gene axis is altered in metabolic disease. Analysis of liver transcriptomic datasets (GSE126848) demonstrated significant downregulation of key insulin clearance-related genes, including *CEACAM1* (Figure 4A), *INSR* (Figure 4B), and *IDE* (Figure 4C) in MASLD patients.

Similarly, core circadian regulators, including *NR1D1* (Figure 4F), *NR1D2* (Figure 4G), *PER1* (Figure 4H), *PER2* (Figure 4I) and *CRY2* (Figure 4K) were also significantly reduced, while there was no significant difference in the transcriptomic expression of *CLOCK* (Figure 4D), *BMAL1* (Figure 4E) and *CRY1* (Figure 4J) between MASLD and controls.

**Figure 5:**
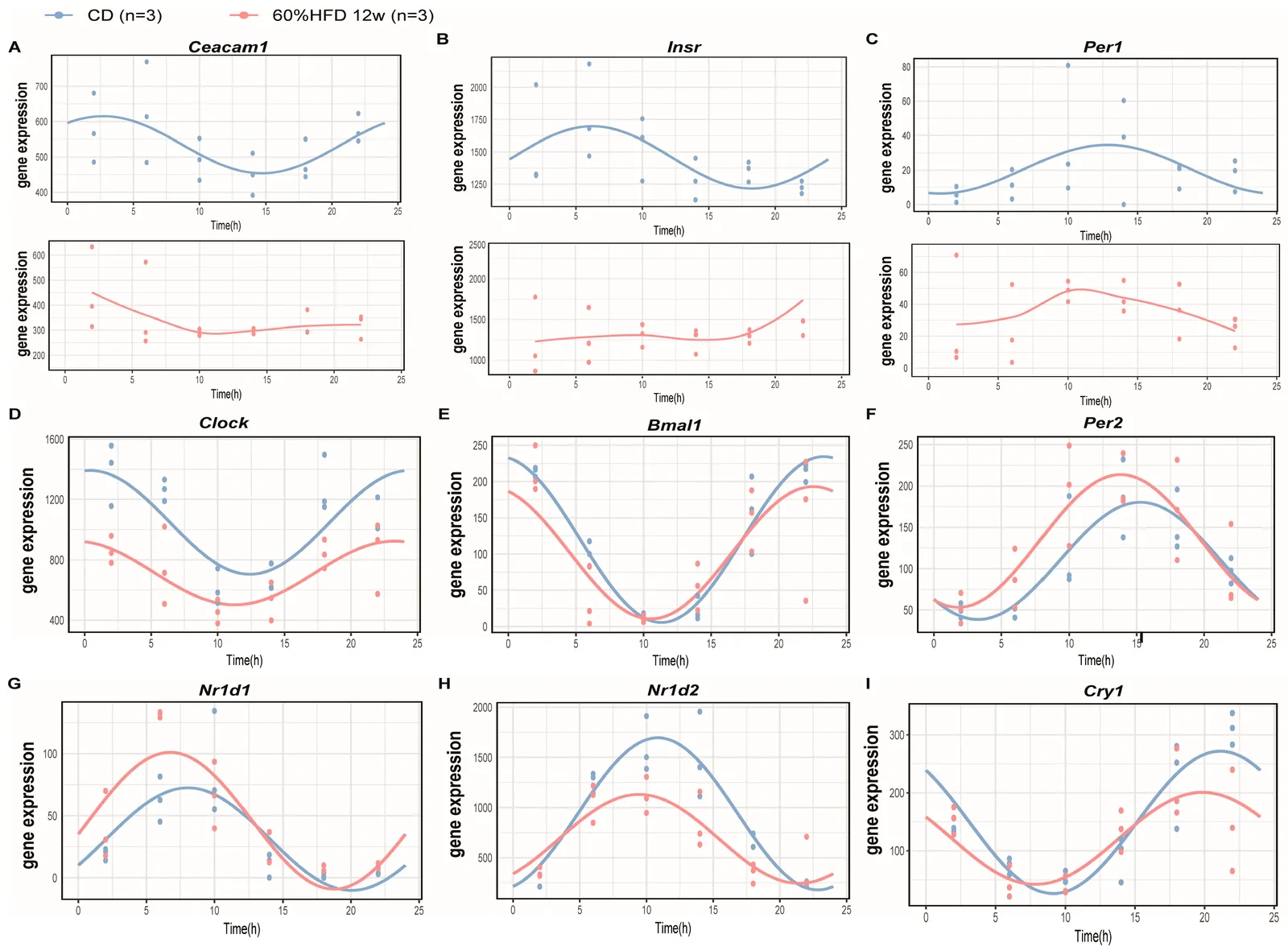
Altered circadian rhythmicity in insulin clearance-related genes under metabolic stress. Time course transcriptomic analysis of CD and 60% HFD 12-week mice (GSE278920) showed disturbed rhythmic expression of genes involved in insulin clearance *Ceacam1* (**A**), *Insr* (**B**) and circadian rhythmicity genes *Per1* (**C**), *Clock* (**D**), *Bmal1* (**E**), *Per2* (**F**), *Nr1d1* (**G**), *Nr1d2* (**H**), *Cry1* (**I**) in HFD mice compared to controls. Oscillation patterns were analyzed for multiple time points.

In addition, analysis of liver time course transcriptomic data from CD and HFD mice (GSE278920) showed that the circadian rhythms of *Ceacam1* (Figure 5A), *Insr* (Figure 5B), and *Per1* (Figure 5C) were abolished in HFD mice compared with controls, while other genes including *Clock* (Figure 5D), *Bmal1* (Figure 5E), *Per2* (Figure 5F), *Nr1d1* (Figure 5G), *Nr1d2* (Figure 5H) and *Cry1* (Figure 5I) remained similar circadian rhythms patterns.

Together, these findings suggest that the coordinated regulation of insulin clearance and circadian gene expression is disrupted under metabolic stress conditions.

## 4. Discussion

In this study, we integrated a single-subject pilot assessment, public hyperinsulinemic-euglycemic clamp datasets, circadian transcriptomic datasets, MASLD liver expression profiles, spatial transcriptomic data, and a time course HFD mouse model to explore the relationship between diurnal insulin clearance and hepatic circadian–metabolic gene signatures. The main findings were as follows: first, the pilot assessment suggested higher evening than morning meal test–derived insulin clearance, and a similar daytime–nighttime pattern was observed in independent clamp datasets; second, selected insulin clearance-related and circadian genes showed time-dependent expression patterns in public circadian transcriptomic datasets; third, MASLD-associated datasets showed reduced expression and altered spatial distribution of insulin clearance-related and circadian gene signatures; and fourth, rhythmic expression of selected genes was attenuated in HFD mouse model under metabolic stress. Together, these findings support a potential link between diurnal insulin clearance and circadian–metabolic gene programs in MASLD, while emphasizing the need for validation in larger controlled human studies.

### 4.1. Diurnal Variation in Insulin Clearance

Insulin clearance is a major determinant of circulating insulin availability, yet its temporal regulation remains less well characterized than insulin secretion or insulin sensitivity [1]. Previous physiological and clinical studies have shown that glucose regulation, insulin secretion, insulin sensitivity, and insulin clearance may vary across the day–night cycle [14,15,16]. Our pilot observation suggested that meal test–derived insulin clearance may be higher at the evening time point than at the morning time point. This observation reflects a difference in a surrogate insulin clearance index rather than a direct measurement of hepatic insulin extraction. Although this single-subject finding should be interpreted as exploratory, the direction of change was supported by independent hyperinsulinemic-euglycemic clamp datasets, in which insulin clearance was also relatively higher during the nighttime or resting phase.

These findings are consistent with the concept that insulin clearance may not be a static hepatic function but rather a dynamic component of temporal metabolic regulation. In MASLD, recent clinical evidence has suggested that reduced nocturnal insulin availability may be related to both altered insulin secretion and increased insulin clearance [8]. Our results extend this concept by integrating multi-dataset physiological and transcriptomic profiling evidence, suggesting that diurnal insulin clearance may be linked to broader circadian–metabolic organization. However, because the pilot experiment included only one healthy participant and two time points, the current study cannot establish an endogenous circadian rhythm of insulin clearance. Controlled studies with larger sample sizes, repeated sampling across 24 h, and standardized light, feeding, sleep, and activity conditions are needed to distinguish true circadian regulation from behavioral and environmental time-of-day effects [16].

### 4.2. Circadian Metabolic Gene Signatures Associated with Insulin Clearance

To explore molecular correlates of diurnal insulin clearance, we analyzed predefined genes involved in insulin clearance and circadian regulation. Insulin clearance-related genes, including *CEACAM1*, *INSR*, and *IDE*, are biologically plausible candidates because they participate in insulin receptor signaling, receptor-mediated insulin uptake, and insulin degradation [1,2]. Core circadian regulators, including *CLOCK*, *BMAL1(ARNTL)*, *NR1D1*, *NR1D2*, *PER1*, *PER2*, *CRY1* and *CRY2*, provide a transcriptional framework through which hepatic metabolic pathways may be temporally organized [14,17].

In public circadian transcriptomic datasets, selected genes showed time-dependent expression patterns, supporting the possibility that insulin clearance–related pathways may be temporally coordinated with systemic circadian programs. Among these genes, *CEACAM1* is particularly relevant because of its established role in hepatic insulin extraction and receptor-mediated insulin internalization [1,2]. *IDE* may further contribute to intracellular insulin degradation, whereas *INSR* links insulin binding and receptor trafficking to downstream metabolic signaling [1]. The observed temporal expression patterns of selected circadian genes suggest that insulin clearance-related pathways may be coordinated with broader circadian–metabolic programs.

These results should be interpreted cautiously. The human circadian transcriptomic datasets used in this study were derived from peripheral blood rather than liver tissue and therefore serve as systemic circadian proxy datasets, not direct measurements of hepatic circadian expression. Therefore, these datasets provide information on systemic circadian timing rather than liver-specific clock activity. In addition, this study failed to perform individual-level paired physiological and liver transcriptome correlation analysis and therefore did not enable the establishment of a causal regulatory hypothesis of circadian genes on insulin clearance.

### 4.3. Relevance to MASLD and Nocturnal Metabolic Dysfunction

MASLD is increasingly recognized as a disease with pronounced temporal metabolic features. Recent clinical work has shown that metabolic dysfunction in MASLD may be accentuated during the nighttime or resting phase, including alterations in hepatic and peripheral insulin sensitivity, de novo lipogenesis, substrate utilization, and insulin availability [8]. Human liver transcriptomic and metabolomic analyses have also shown that time-of-day-dependent molecular programs are disrupted during MASLD progression to MASH, although core molecular clock differential expression may be preserved [18]. Within this context, insulin clearance may be particularly relevant because changes in hepatic insulin extraction could influence systemic insulin exposure, nocturnal substrate handling, and compensatory beta-cell demand.

Our integrative analyses suggest that MASLD is associated with alterations in both insulin clearance–related genes and circadian regulatory genes. In MASLD liver datasets, selected genes involved in insulin clearance and circadian regulation showed reduced expression compared with controls. Spatial transcriptomic analysis further provided a spatial framework for interpreting the distribution of these signatures within the hepatic microenvironment, suggesting regional differences in their localization patterns. This interpretation is consistent with recent spatially resolved multi-dataset work demonstrating marked spatial and microenvironmental remodeling in human MASLD liver tissue [9]. In the HFD mouse model, time-dependent expression of selected genes was attenuated under metabolic stress, supporting the possibility that metabolic injury may disrupt temporal organization of hepatic insulin clearance-related pathways.

Taken together, these findings support a model in which MASLD-related metabolic stress may be associated with altered temporal regulation and spatial distribution patterns of insulin clearance-related pathways. Rather than representing a uniform static defect, insulin clearance may vary across the day–night cycle and may interact with hepatic circadian–metabolic programs. This model is consistent with the observed nocturnal metabolic vulnerability in MASLD, but it remains hypothesis-generating. Direct human liver sampling, interventional circadian studies, and mechanistic experiments are needed to determine whether altered circadian–metabolic gene programs causally influence hepatic insulin clearance in MASLD.

### 4.4. Clinical and Translational Implications

The potential diurnal variation in insulin clearance has several clinical implications. First, time-dependent differences in insulin clearance may influence circulating insulin exposure, glycemic regulation, and interpretation of dynamic metabolic tests. Second, in MASLD, altered nighttime insulin availability may contribute to impaired metabolic flexibility and dysregulated lipid handling during the resting phase. Third, if confirmed in larger studies, insulin clearance could become an additional temporal phenotype for understanding heterogeneity in insulin resistance, hyperinsulinemia, and MASLD progression.

These findings also highlight the relevance of chronobiology for metabolic disease research. Meal timing, sleep regularity, physical activity, and pharmacological timing may influence insulin secretion, insulin sensitivity, and insulin clearance in different ways [14,17,19]. However, the current study does not test chronotherapeutic interventions and should not be interpreted as evidence that targeting circadian pathways will improve insulin clearance or MASLD outcomes. Future studies should evaluate whether interventions that align feeding and activity with endogenous circadian rhythms can modify insulin clearance dynamics and improve metabolic health in patients with MASLD.

### 4.5. Limitations

Several limitations should be acknowledged. First, the human pilot assessment included only one healthy participant and two time points; therefore, it should be interpreted as a proof-of-concept observation rather than evidence of a generalizable or endogenous circadian rhythm of insulin clearance. Larger controlled studies with repeated sampling across 24 h are needed to distinguish intrinsic circadian regulation from behavioral and environmental time-of-day effects, including feeding, sleep, light exposure, and physical activity.

Second, the meal test-derived insulin clearance surrogate index reflects the relative relationship between C-peptide and insulin exposure during a meal challenge and does not directly quantify hepatic insulin extraction. Although clamp-derived metabolic clearance rates were analyzed from independent datasets, these two approaches represent different aspects of insulin clearance physiology and should not be considered interchangeable.

Third, the integrative multi-dataset analyses relied on public datasets that differed in species, tissue source, disease definition, sampling design, and data-processing procedures. Human peripheral blood transcriptomic datasets were used as systemic circadian proxies and cannot substitute for direct human liver circadian profiling. The dataset included a small number of populations, and the differences in spatial distribution suggested by the spatial transcriptomic analysis still warrant quantitative confirmation in a larger dataset. Thus, the current findings remain hypothesis-generating and require expansion of the cohort to be validated by direct human studies and mechanistic experiments.

In addition, physiological insulin clearance measurements and transcriptomic datasets were derived from independent cohorts. Therefore, the current study does not establish individual-level molecular associations between circadian gene expression and insulin clearance.

## 5. Conclusions

In conclusion, this integrative analysis suggests that insulin clearance may exhibit diurnal variation and may be linked to hepatic circadian–metabolic gene signatures altered in MASLD. By combining a single-subject pilot assessment with public clamp datasets and multi-dataset resources, this study provides a hypothesis-generating framework for understanding insulin clearance as a temporally regulated metabolic phenotype. Larger controlled human studies and mechanistic experiments are warranted to validate the temporal regulation of insulin clearance and clarify its relevance to MASLD pathogenesis and metabolic intervention.

## Data Availability

The original contributions presented in this study are included in the article/Supplementary Materials. Further inquiries can be directed to the corresponding authors. Other original data from public datasets presented in the study are openly available and summarized in Table S1.

## Supplementary Materials

The following supporting information can be downloaded at: https://www.mdpi.com/article/10.3390/metabo16090629/s1, Table S1: Public dataset information.

## Abbreviations

The following abbreviations are used in this manuscript:

*BMAL1*	Basic helix-loop-helix ARNT like 1
*CEACAM1*	Carcinoembryonic antigen-related cell adhesion molecule 1
*CLOCK*	Clock circadian regulator
*CRY1*	Cryptochrome 1
*CRY2*	Cryptochrome 2
HEC	Hyperinsulinemic euglycemic clamp
HFD	High fat diet
ICR	Insulin clearance rate
*IDE*	Insulin degrading enzyme
*INSR*	Insulin receptor
MASLD	Metabolic dysfunction-associated steatotic liver disease
*NR1D1*	Nuclear receptor subfamily 1 group D member 1
*NR1D2*	Nuclear receptor subfamily 1 group D member 2
*PER1*	Period circadian regulator 1
*PER2*	Period circadian regulator 2
ZT	Zeitgeber time
